# *LEAFY COTYLEDON2* (*LEC2*) promotes embryogenic induction in somatic tissues of Arabidopsis, via *YUCCA*-mediated auxin biosynthesis

**DOI:** 10.1007/s00425-013-1892-2

**Published:** 2013-05-31

**Authors:** Barbara Wójcikowska, Karolina Jaskóła, Przemysław Gąsiorek, Magdalena Meus, Katarzyna Nowak, Małgorzata D. Gaj

**Affiliations:** Department of Genetics, University of Silesia, ul. Jagiellońska 28, 40-032 Katowice, Poland

**Keywords:** *Arabidopsis*, Auxin biosynthesis, *LEC2*, Somatic embryogenesis, *YUCCA*

## Abstract

The *LEAFY COTYLEDON2* (*LEC2*) transcription factor with a plant-specific B3 domain plays a central role in zygotic and somatic embryogenesis (SE). LEC2 overexpression induced in planta leads to spontaneous somatic embryo formation, but impairs the embryogenic response of explants cultured in vitro under auxin treatment. The auxin-related functions of *LEC2* appear during SE induction, and the aim of the present study was to gain further insights into this phenomenon. To this end, the effect of LEC2 overexpression on the morphogenic responses of *Arabidopsis* explants cultured in vitro under different auxin treatments was evaluated. The expression profiles of the auxin biosynthesis genes were analysed in embryogenic cultures with respect to LEC2 activity. The results showed that LEC2 overexpression severely modifies the requirement of cultured explants for an exogenous auxin concentration at a level that is effective in SE induction and suggested an increase in the auxin content in 35S::LEC2-GR transgenic explants. The assumption of an LEC2 promoted increase in endogenous auxin in cultured explants was further supported by the expression profiling of the genes involved in auxin biosynthesis. The analysis indicated that *YUCCAs* and *TAA1,* working in the IPA-YUC auxin biosynthesis pathway, are associated with SE induction, and that the expression of three *YUCCA* genes (*YUC1, YUC4* and *YUC10*) is associated with LEC2 activity. The results also suggest that the IAOx-mediated auxin biosynthesis pathway involving *ATR1/MYB34* and *CYP79B2* does not seem to be involved in SE induction. We conclude that de novo auxin production via the tryptophan-dependent IPA-YUC auxin biosynthesis pathway is implicated in SE induction, and that *LEC2* plays a key role in this mechanism.

**Electronic supplementary material:**

The online version of this article (doi:10.1007/s00425-013-1892-2) contains supplementary material, which is available to authorized users.

## Introduction

Unlike the spectacular progress that have been made in identifying the key genetic factors that control the reprogramming of somatic cells in animals (Wang et al. 2011), the molecular mechanisms involved in the determination of plant tissue developmental plasticity are poorly understood. Only recently, the molecular events governing embryogenic induction in somatic plant cells begun to be decoded, and with the use of global transcriptome analysis many genes have been shown to be involved in the somatic embryogenesis (SE) that is induced in different plant species (Thibaud-Nissen et al. 2003; Sharma et al. 2008; Imin et al. 2008; Lucau-Danila et al. 2010). However, very few genes have been experimentally confirmed to be SE-specific and even fewer have been elucidated in terms of their mechanism of action during SE induction. Most of the genes to date have been recognised as SE-promoting encode transcription factors, including *BABY BOOM* (*BBM*, Boutilier et al. 2002), *WUSCHEL* (*WUS*, Zuo et al. 2002), *AGAMOUS*-*LIKE15* (*AGL15*, Harding et al. 2003), *LEAFY* *COTYLEDON* (*LEC,* Gaj et al. 2005), *AtMYB115*, *AtMYB118* (Wang et al. 2009) and *EMBRYO MAKER* (Tsuwamoto et al. 2010).

The *LEC2* of the *LEC* group of genes encodes a transcription factor with a plant-specific B3 DNA binding motif and plays a master regulatory function in *Arabidopsis* embryo and seed development (Braybrook and Harada 2008). Loss-of-function mutations in *LEC2* lead to pleiotropic effects, including the formation of leafy cotyledons, i.e. cotyledons with trichomes on the adaxial surface, defects in suspensor morphology during early embryogenesis and desiccation-intolerant seeds that have to be rescued before seed maturation (Meinke et al. 1994). *LEC2* directly triggers the expression of seed-specific genes (Santos-Mendoza et al. 2005) and controls oil and protein metabolism in maturing seeds (Kroj et al. 2003; Baud et al. 2007). Recent analysis has indicated that LEC2 may also be involved in carbon partitioning toward different storage compounds, such as oil, proteins, and carbohydrates (Angeles-Núnez and Tiessen 2011). *LEC2* is expressed in zygotic embryos between 4 and 14 days after pollination in a developmentally regulated pattern (Stone et al. 2001; Kroj et al. 2003), and different genetic factors repress its expression in post-embryogenic tissue, including PICKLE (PKL), a chromatin remodeler (Ogas et al. 1999) and fertilisation-independent endosperm (FIE), the *Arabidopsis* homolog of the PRC2 complex (Bouyer et al. 2011).

In addition to the versatile regulatory functions of *LEC2* in zygotic embryogenesis and seed development, the gene has been implicated in promoting the embryogenic response of somatic tissue. It was reported that overexpression of LEC2 triggers spontaneous somatic embryo formation in plants (Stone et al. 2001), while a mutation in this gene impairs the embryogenic response of explants cultured in vitro (Gaj et al. 2005).

Considering auxin as a key factor triggering SE induction in *Arabidopsis* and other plants (Gaj 2004), an auxin-related mechanism was proposed as the most likely process involved in the *LEC2*-mediated control of embryogenic response in *Arabidopsis* tissue (Stone et al. 2008). In line with this idea, auxin response *INDOLE*-*3*-*ACETIC ACID INDUCIBLE30* (*IAA30*) and auxin biosynthesis *YUCCA2* (*YUC2*) and *YUCCA4* (*YUC4*) were associated with the LEC2-controlled genes (Braybrook et al. 2006; Stone et al. 2008). In addition, a close link between *LEC2* activity and exogenously applied auxin was indicated (Ledwoń and Gaj 2009). The gene was shown to be up-regulated in an embryogenic culture induced in vitro on an auxin medium, and the overexpression of LEC2 was found to compensate for the auxin treatment as somatic embryo formation was observed in explants cultured under auxin-free conditions (Ledwoń and Gaj 2009). Together, these observations lead to the hypothesis that LEC2 may influence the embryogenic potential of somatic tissue through the control of the level of endogenous auxin.

In the present study, to gain insight into the auxin-related mechanisms involved in the LEC2 control of SE, in vitro morphogenic responses of tissues overexpressing LEC2 were evaluated in relation to the auxin type and concentration used in the culture medium. The expression patterns of auxin biosynthesis genes involved in two alternative tryptophan-dependent pathways were profiled during SE in relation to LEC2 activity. The results have provided further evidence that *LEC2* influences the embryogenic response of cultured somatic tissue through the activation of the *YUC*s (*YUC1, 4* and *10*) of the IPA-YUC auxin biosynthesis pathway, which possibly results in local auxin production in somatic tissue.

## Materials and methods

### Plant material

The Columbia (Col-0) genotype of *Arabidopsis thaliana* (L.) Heynh., the transgenic plants overexpressing the *LEC2* (35S::LEC2-GR, Col-0), and the *yucca2* and *yucca4* insertional mutants were used. The 35S::LEC2-GR (12/1/8) line harboured a single copy of a transgene and displayed a high and stable level of the *LEC2* transcript under DEX treatment (Ledwoń and Gaj 2009). Seeds of the Col-0 parental genotype and the insertional mutants, *yuc2* (SALK_030199; N659779) and *yuc4* (SALK_047083; N668198), were supplied by NASC (The Nottingham Arabidopsis Stock Centre, UK). Insertions carried by the *yuc2* and *yuc4* mutants were shown to abolish the transcription of the respective *YUC2* and *YUC4* gene (Supplemental Fig. S1).

### Induction of LEC2 function in transgenic plants

In order to control the activity of the LEC2 protein, a system of posttranslational activation was applied (Sablowski and Meyerowitz 1998) in which a fusion the LEC2-GR protein with the hormone-binding domain of the rat glucocorticoid receptor (GR) enters the nucleus upon induction with the mammalian steroid hormone analogue, dexamethasone (water soluble DEX, Sigma Cat# D2915). DEX was added to the media at a concentration of 30 μM to activate the LEC2 protein in transgenic explants and seedlings.

### Explants for in vitro culture

Immature zygotic embryos (IZEs) in the late cotyledonary stage of development and fragments (roots, hypocotyls, petioles and cotyledons) of 14-day-old seedlings of the Col-0 and 35S::LEC2-GR transgenic line were used as explants for in vitro cultures. IZE explants were isolated and sterilised according to a standard procedure (Gaj 2001). To produce explants from seedlings, seeds were sterilised in a 20 % solution of commercial bleach for 20 min and then rinsed thrice in sterile water. Seeds were then germinated on a half-concentrated MS medium (Murashige and Skoog 1962) containing 10 g L^−1^ sucrose and 8 g L^−1^ agar. Ten explants were cultured in one Petri dish, and thirty explants, in three replicates, were analysed in each culture combination.

### Plant growth and in vitro culture conditions

Plants used for the analysis of indolic compounds and as a source of IZE explants were grown in a mixture of soil and vermiculite (1:1) at 22 °C under a 16-h photoperiod of 100 μM photons m^−2^ s^−1^ of white, fluorescent light. Plant materials grown under sterile conditions were kept at 23 °C under a 16-h photoperiod of 40 μM m^−2^ s^−1^ white, fluorescent light.

### Somatic embryogenesis

A standard protocol was used to induce somatic embryos (Gaj 2001). Ten IZEs were cultured in a Petri dish (35 mm) on a Phytagel-solidified (3.5 g L^−l^) induction medium containing basal B5 micro and macro-elements (Gamborg et al. 1968) and 20 g L^−1^ sucrose. A standard auxin (E5) induction medium with 5 μM 2,4-dichlorophenoxyacetic acid (2,4-D; Sigma) and an auxin-free (E0) medium were used. In some experiments, modifications of the auxin content in the induction medium were applied, including various concentrations of 2,4-D over a range of 0.1–50 μM, and the replacement of 2,4-D with indole-3-acetic acid (IAA) at 30, 40 and 50 μM and 1-naphthalene acetic acid (NAA) at 5, 10, 20 μM. Explants, IZEs and seedling fragments (hypocotyls, petioles and cotyledons) were cultured and their embryogenic potential was evaluated in respect to the auxin content in the medium and the LEC2 expression level.

### Shoot organogenesis

To induce shoot formation via organogenesis (ORG), IZEs and roots isolated from 2-week-old seedlings were cultured. Explants were incubated for 7 days in a liquid callus induction medium (CIM), followed by a culture on solid shoot induction media (SIM). The standard CIM medium contained a basal composition of B5 medium (Gamborg et al. 1968), 0.5 g L^−1^ MES, 20 g L^−1^ glucose, 2.2 μM of 2,4-D and 0.2 μM of kinetin (Feldmann and Marks 1986). Shoot formation in cultures of the CIM-treated seedling roots and IZE-derived cotyledons was induced on SIM-R (Feldmann and Marks 1986) and SIM-C (Kraut et al. 2011) regeneration media, respectively. The SIM-R medium was composed of a basal B5 medium (Gamborg et al. 1968) and supplemented with 0.5 g L^−1^ MES, 20 g L^−1^ glucose, 5 μM of 6-(α,α-dimethylallylamino)-purine (2iP) and 0.8 μM of indole-3-acetic acid (IAA), according to Feldmann and Marks (1986). The SIM-C medium contained micro-elements of MS (Murashige and Skoog 1962), macro-salts and vitamins of a B5 medium (Gamborg et al. 1968) and was supplemented with 30 g L^−1^ sucrose, 0.5 μM of NAA and 4.4 μM of BAP (6-benzylaminopurine) (Kraut et al. 2011). Modifications of the standard CIM and SIM media included a 10× increase of the auxin concentration to 22 μM 2,4-D in CIM, 8 μM IAA in SIM-R and 5 μM NAA in SIM-C.

### Evaluation of the culture morphogenic capacity

The capacity for SE and ORG was evaluated in 3-week-old cultures. Two parameters of culture morphogenic potential were evaluated: SE/ORG efficiency as the frequency of the explants producing somatic embryos/shoots and SE/ORG productivity defined as the average number of somatic embryos/shoots developed per explant.

### Content of indolic compounds

A colorimetric technique that enabled the detection of indolic compounds, including IAA, was applied (Bric et al. 1991). To verify the reliability of this technique for the analysis of plant tissue, the tissues of Col-0 plants with significantly different IAA levels were analysed. The analysis involved the roots and leaves of 10 and 17 DAG seedlings, respectively (Kowalczyk and Sandberg 2001), and the leaves of different ages isolated from 4-week-old plants: young *versus* old rosette leaves (Graaff et al. 2003). To evaluate the relationship between IAA content and LEC2 activity, 4-week-old seedlings and IZE-derived cultures of Col-0 and 35S::LEC2-GR genotypes were analysed.

Seedlings were grown from surface-sterilised seeds (a 20 % solution of commercial bleach for 20 min) on a MS agar basal medium (Murashige and Skoog 1962). IZE explants were induced on an E5 medium and tissues were sampled at the 5th and 21st days of the culture. In addition, to the 21-day-old Col-0 cultures developing somatic embryos, the explants that failed in SE induction and produced a non-embryogenic callus were analysed.

Fresh tissue (10 and 100 mg from an in vitro culture and plants/seedlings, respectively) was transferred immediately after harvest to mortars containing 2 mL of 10× PBS and kept at 4 °C. The material was homogenised and the solution centrifuged (25 min; 18,000×*g*). 2 mL of supernatant was mixed with 100 μL of 10 mM orthophosphoric acid and 4 mL of Salkowski’s reagent (150 mL H_2_SO_4_; 250 mL ddH_2_O; 7.5 mL 0.5 M FeCl_3_ × 6H_2_O). The absorbance of a pink colour developed after a 30-min incubation at room temperature was read at 530 nm. The IAA concentration was determined using a calibration curve of pure IAA (solutions of IAA were freshly prepared in 10× PBS), as the standard following linear regression analysis. Each analysis was carried out in three replicates.

### RNA isolation and RT-qPCR analysis

An RNAqueous Kit (Ambion) was used to isolate total RNA from the IZE explants induced on an auxin (E5) medium at 0, 3, 5, 10 and 15 days. Depending on the age of the culture, from 250 (0 d) to 4 (15 d), explants were then used for RNA isolation. The concentration and purity of RNA was evaluated with a ND-1000 spectrophotometer (NanoDrop). In order to control DNA contamination, the RNAs were treated with RQ1 RNase-free DNase I (Promega) following the manufacturer’s instructions. First-strand cDNA was produced in a 20-μL reaction volume using the RevertAid First Strand cDNA Synthesis Kit (Fermentas). The product of reverse transcription was diluted with water at a 1:1 ratio, and 1 μL of this solution was used for RT-PCR or RT-qPCR reactions. RT-qPCR was carried out in a 10-μL reaction volume using a LightCycler Fast- Start DNA Master SYBR Green I (Roche).

The LightCycler 2.0 (Roche) real-time detection system was used under the following reaction conditions: denaturation one repeat of 10 min at 95 °C, followed by 45 repeats of 10 s at 95 °C, 8 s at 55 °C, 12 s at 72 °C and 5 s at 80 °C. Denaturation for melt curve analysis was conducted at 95 °C followed by 15 s at 65 °C and 95 °C (0.1 °C/s for fluorescence measurement).

Primary data analysis was performed using LightCycler Software 4.0 (Roche). Relative RNA levels were calculated and normalised to an internal control, the *At4g27090* gene encoded 60S ribosomal protein (Thellin et al. 1999). In all of the analysed tissue samples, the control gene exhibited a constant expression pattern with Cp = 18 ± 1.

The following primers were used for the expression profiling of the genes analysed:

- *YUC1* pF CGGAACACCGTTCATGTGT pR CCGGTGACATTTTTCAGCTC
- *YUC2* pF TTGTGGTTCGTGACTCGGTA pR TTCAAGAGGGCCAAGTTTTG
- *YUC3* pF GATGGCCGTGTTCTTGAGAT pR TCATGAGCCACACTCATAGC
- *YUC4* pF AACTCCCGTTCTTGATGTCG pR AAAAACTATTCTCCTTAAGCCAATC
- *YUC5* pF TGTCCAGTCTGCTCGATACG pR TTCTCGCCGGATTTGTACTC
- *YUC6* pF GGTAAAACTCCGGTTCTCGAC pR TTGGAAATCCATCTTTCTTACTAAAC
- *YUC7* pF TGAAGAACACCGCAGGTAAA pR CCAAGTCGTTTTCCTTAAGCC
- *YUC8* pF CGTCTCAAGCTTCACCTTCC pR AGCCACTGGTCTCATCGAAC
- *YUC9* pF TGGTCGTTAGAAGCTCGGTT pR CGGCGTCTTTCCTGTCAT
- *YUC10* pF TTACCGGAAAAGCTCCTGTC pR TCACGTATTCATAGTCCTCTAACCA
- *YUC11* pF GAGAATGGCGAAGGTGTGAT pR TAACACGTGCACCTGGCTAC
- *TAA1* pF TTCGTGGTCAATCTGGATCATGG pR ACCACGTATCGTCACCGTACAC
- *CYP79B2* pF TCAAACCCACCATTAAGGAGCTTG pR GACTCTGTCGATCTCTTCCATTGC
- *ATR1/MYB34* pF TAACACGTGCACCTGGCTAC pR CAATGTGGAGGTCGGAGAAT
- *At4g27090* pF GTCGTTATCGTCGACGTTGTT pR CCTCGATCAAAGCCTTCTTCT

The plant tissues for the analysis of gene expression were produced in three biological replicates, and two technical replicates of each repetition were carried out.

### Data presentation and statistical analysis

The averages with standard deviation are presented in Figs. 2–6. To calculate the significant differences (at *P* = 0.05) between the samples compared, the Kruskal–Wallis ANOVA rank was applied and the medians are presented in Table 1, Fig. 7 and Fig. S2, accordingly.

**Table 1:** Endogenous level of indolic compounds (μg/g of fresh tissue) in seedlings and IZE-culture of Col-0 and 35S::LEC2-GR transgenic line with DEX-induced LEC2-overexpression

		Col-0	35S::LEC2-GR - DEX	35S:LEC2-GR + DEX
Seedlings	28 days	65.83 ± 39.58	67.92 ± 31.00	165.01 ± 58.77*

	5 days	240.30 ± 23.93	228.46 ± 39.34	307.18 ± 30.67*
IZE-derived culture				
	21 days	Col-0		
		Somatic embryos	Callus	
		214.48 ± 33.00	292.59 ± 18.09*	

IZE explants were cultured on auxin medium (E5)

* Values significantly different from DEX-free cultures

## Results

### LEC2 overexpression and morphogenic responses in vitro

#### Somatic embryogenesis

In a previous study (Ledwoń and Gaj 2009), we found that LEC2 overexpression induced in an IZE-culture disturbs embryogenic response and on an E5 auxin medium that is efficient for SE induction in WT explants, a callus is produced instead of the direct development of somatic embryos. In addition, an auxin-free medium that is inefficient for SE induction in a WT culture led to somatic embryo production upon LEC2 overexpression. These observations suggested a relation between the LEC2 expression and auxin treatment required for IZE explants to induce SE.

**Fig. 1:**
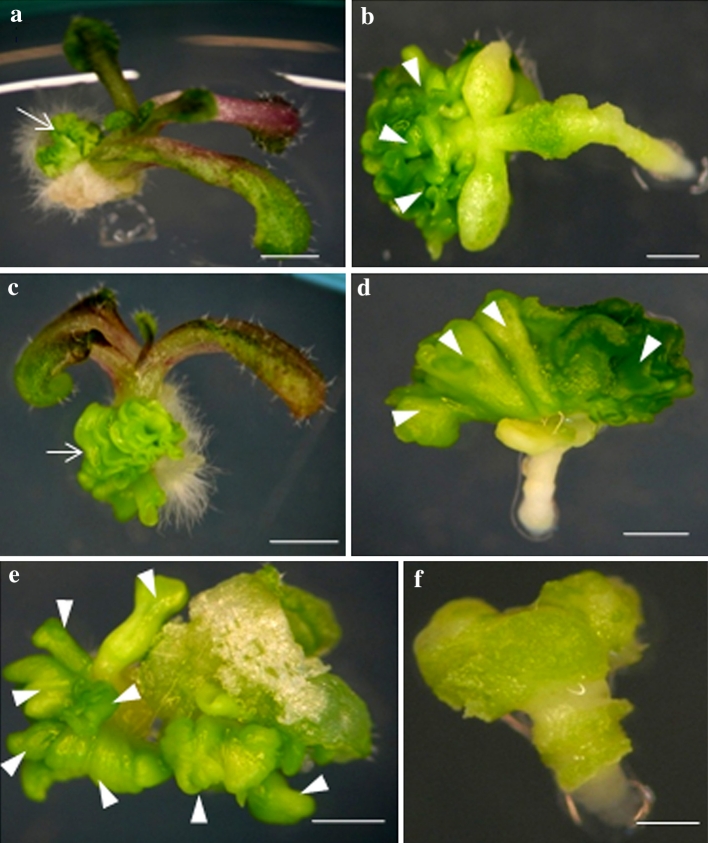
Morphogenic responses of 35S::LEC2-GR IZE explants cultured in the absence (**a, c, e**) or presence (**b, d, f**) of DEX on induction media supplemented with various concentrations of 2,4-D: 0.1 (**a, b**), 1 (**c, d**) and 5 μM (**e, f**). Somatic embryo-like structures on an IZE-developed seedling (**a, c**); numerous somatic embryos (**b, d, e**) and a non-embryogenic callus (**f**). Somatic embryo-like structures (*arrow*) and somatic embryos (*arrow head*) are indicated. *Scale bar* 1 mm

To explore this hypothesis, transgenic IZE explants (35S::LEC2-GR) were cultured in a range of 2,4-D concentrations, and morphological observations revealed that depending on the LEC2 expression level (DEX-treated *versus* DEX-free cultures), the explants displayed drastically different capacities for SE (Fig. 1). In the presence of a low 2,4-D concentration (0.1–1.0 μM), explants not treated with DEX frequently developed into seedlings (Fig. 1a, c), while those overexpressing LEC2 produced somatic embryos (Fig. 1b, d). A standard concentration of 2,4-D (5 μM) in the induction medium resulted in the inhibition of embryogenic response in transgenic DEX-treated explants and a callus was produced instead (Fig. 1f).

**Fig. 2:**
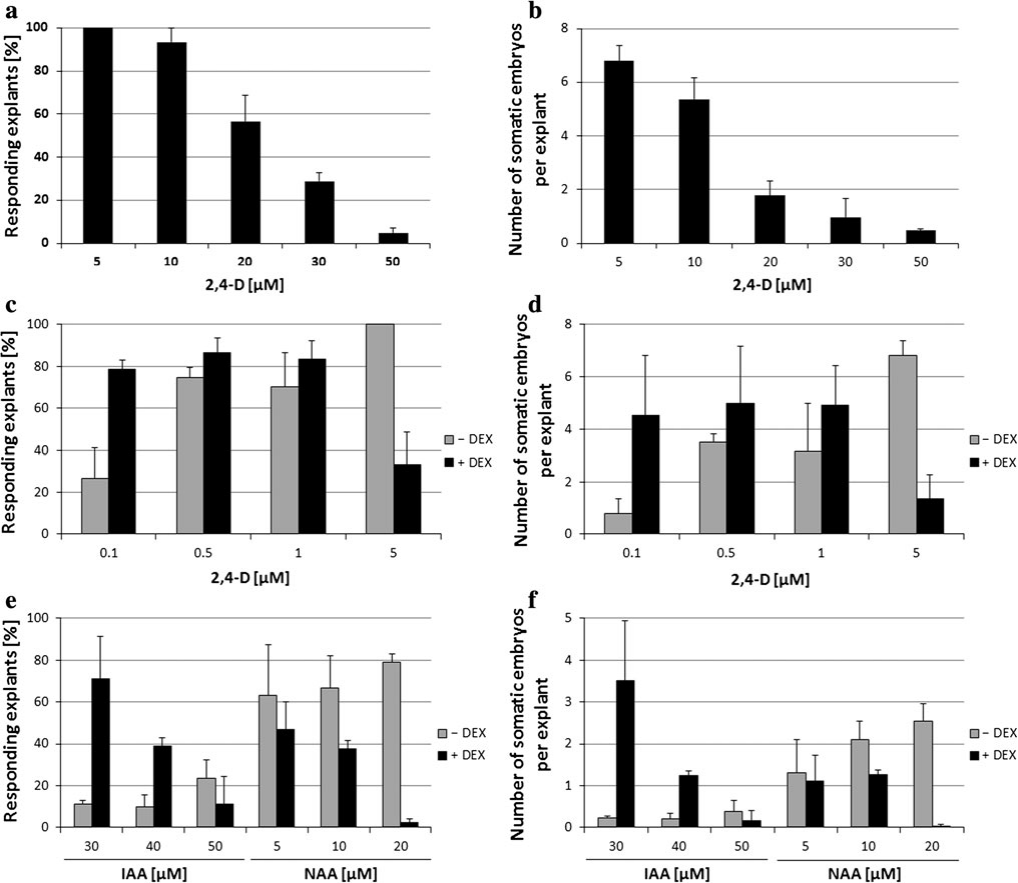
SE efficiency (**a, c, e**) and productivity (**b, d, f**) of Col-0 (**a**, **b**) and 35S::LEC2-GR (**c, d, e, f**) on cultures derived from IZE explants induced under different auxin treatments: 2,4-D (**a**–**d**) or IAA and NAA (**e, f**). DEX treatment (+DEX) was used to induce LEC2 overexpression in transgenic explants. Bars represent standard deviation (*n* = 9)

The responses of LEC2 overexpressing explants were found to mimic WT explants cultured at high 2,4-D concentrations. A decline in the embryogenic capacity of Col-0 explants correlated with increasing 2,4-D concentrations used in the induction medium (Fig. 2a, b). Consequently, 50 μM of 2,4-D almost totally inhibited the embryogenic response of WT explants and efficiently promoted tissue re-differentiation into callus, as was observed in the LEC2-overexpressing explants cultured at a 10× lower (5 μM) 2,4-D concentration. The explants overexpressing LEC2 showed a high capacity for SE induction under a low (0.1 μM) 2,4-D concentration, which was suboptimal for the WT culture (Fig. 2c, d). To reveal whether the observed hypersensitivity of LEC2-overexpressing cultures to auxin was specific for 2,4-D treatment, two other auxins, IAA and NAA, were tested. In a similar way to 2,4-D treated explants, LEC2-overexpression significantly changed the requirements of the explants for IAA and NAA concentrations that were efficient for SE induction (Fig. 2e, f). IAA in concentrations of 30–50 μM was inefficient for SE induction in the control culture (DEX-free), and a maximum 25 % of the explants developed somatic embryos. In contrast, the DEX-treated explants displayed a high SE efficiency (over 70 %) and productivity in the presence of a lower IAA (30 μM) concentration. Similarly, explants overexpressing LEC2 showed an increased sensitivity to NAA, and the most efficient concentration for SE induction in DEX-free culture, 20 μM, almost totally inhibited embryogenic response in the LEC2-overexpressing culture and promoted callus formation.

**Fig. 3:**
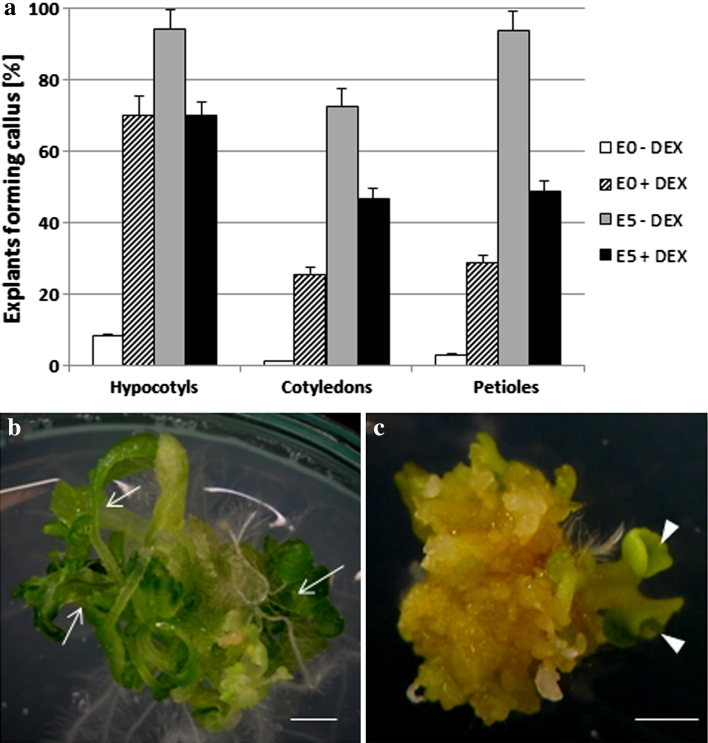
Morphogenic responses of 35S::LEC2-GR seedling explants (hypocotyls, cotyledons and petioles) under DEX-induced (+DEX) LEC2 overexpression on an auxin (E5) and auxin-free (E0) medium. Frequency of explants developing a callus (**a**). Development of shoots (**b**) and somatic embryos (**c**) on an auxin-free (E0) medium. Shoots (a*rrows*) and somatic embryos (*arrow head*) are indicated. Bars represent standard deviation (*n* = 3). *Scale bar* 1 mm

An increased activity of, or sensitivity to, auxin in LEC2 overexpressing tissue was also implied when seedling explants (hypocotyls, cotyledons and petioles) were cultured in auxin and auxin-free media (Fig. 3). Regardless of the explant type that was cultured, LEC2 overexpression promoted callus formation on the auxin-free medium, and in contrast, a significantly reduced callogenesis was observed in the presence of auxin (Fig. 3a). In addition to callus formation, the seedling explants overexpressing LEC2 cultured on an E0 medium displayed shoot formation (Fig. 3b), and somatic embryos were also noticed sporadically (Fig. 3c).

These results showed conclusively that LEC2 overexpression severely modifies the embryogenic responses of different tissue types under auxin treatment. Regardless of the auxin and explant type, the observed responses implied an increased sensitivity to auxin of transgenic tissue.

#### Shoot organogenesis

**Fig. 4:**
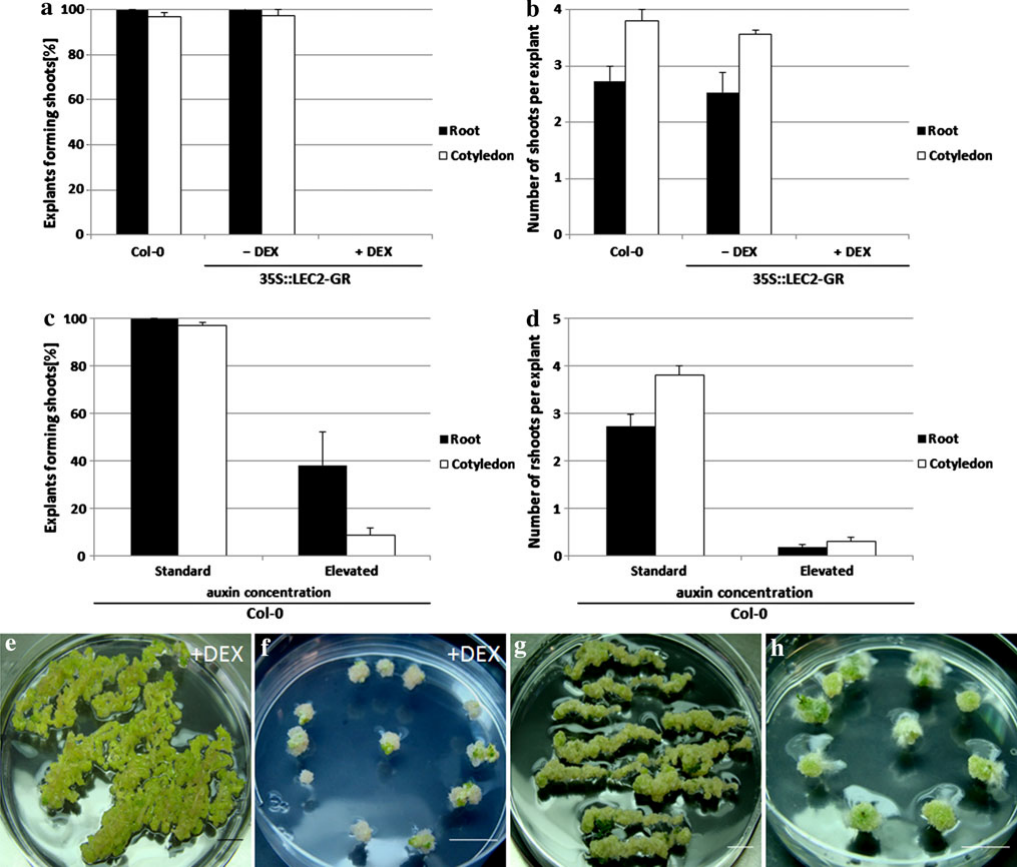
Shoot organogenesis capacity in cultures derived from root and IZE-cotyledon explants of Col-0 and 35S::LEC2-GR (**a**, **b**) and Col-0 (**c**, **d**). A standard (**a**, **b**) and 10× elevated (**c**, **d**) auxin concentration in regeneration media was used and the frequency of shoot-regenerating explants (**a, c**) and the average shoot number regenerated by an explant (**b, d**) were evaluated. Inhibition of shoot regeneration and callus formation in root (**e, g**) and IZE-cotyledon (**f, h**) explants. DEX-treated explants of 35S::LEC2-GR were induced under standard culture conditions (**e, f**) and Col-0 explants were cultured under an elevated auxin concentration (**g, h**). LEC2 overexpression was induced with DEX (+DEX). Bars represent standard deviation (*n* = 9). *Scale bar* 1 cm

Auxin, as a sole phytohormone, is used in *Arabidopsis* to induce SE, while a combination of auxin and cytokinin results in shoot organogenesis. To test whether LEC2 overexpression also modifies the morphogenic response of tissues induced toward shoot regeneration, IZE-cotyledon and seedling-root explants induced on a CIM medium were transferred to cytokinin-enriched SIM media to promote shoot organogenesis. It was observed that a 35S::LEC2-GR transgenic root and IZE-cotyledon explants that were not treated with DEX (-DEX) responded similarly to WT explants (Col-0) and produced shoots on SIM-R and SIM-C media efficiently, respectively. In contrast, transgenic explants induced with DEX (+DEX) were found to be totally unable to undergo shoot regeneration (Fig. 4a, b). Responses similar to those displayed by the LEC2-overexpressing culture were observed in the cultures of Col-0 explants induced on SIM media with a 10× elevated auxin concentration. An increased auxin concentration, regardless of the explant type, resulted in a drastic reduction of explant frequency for regenerating shoots, as well as the average shoot number produced per explant (Fig. 4c, d). Moreover, the transgenic explants treated with DEX and cultured on standard media, and the WT explants induced on media with a 10× elevated 2,4-D concentration, readily produced a callus (Fig. 4e, f, g, h).

#### Indolic compound level in tissue overexpressing LEC2

The analysis of the morphogenic responses of explants overexpressing LEC2 with respect to auxin treatment implies an increased sensitivity to auxin of transgenic tissue. In addition, the transgenic seedlings with DEX-induced LEC2 overexpression were reported to display severe developmental defects, including reduced growth, swollen cotyledons and spontaneous callus and somatic embryo development (Stone et al. 2001; Ledwoń and Gaj 2009). In light of all of these observations, we hypothesised that LEC2 overexpression may result in an increased content of endogenous IAA. To test this assumption, we used a colorimetric method based on the Salkowski reagents (Bric et al. 1991), which enable the detection of indolic compounds correlated with IAA production and which is routinely applied to indicate IAA production in bacteria (Ma et al. 2009; Luo et al. 2011). Our analysis proved that this method may also be useful for a rough estimation of the IAA level in plant tissue. Similar to other reports on IAA content in different tissues of *Arabidopsis* plants (Kowalczyk and Sandberg 2001; Graaff et al. 2003), we found a significantly higher content of indolic compounds in the roots rather than in leaves of seedlings and in the younger rather than older leaves of 4-week-old plants (Supplemental Fig. S2).

This method was then applied to study the relationship between LEC2 expression and auxin level. An analysis of 4-week-old seedlings indicated a more than twofold increase in the content of indolic compound in the 35S::LEC2-GR seedlings overexpressing LEC2 compared with the control (Col-0) and with transgenic tissue that had not been treated with DEX (Table 1). Indolic compounds were also evaluated in transgenic explants cultured for 5 days on an E5 medium. The results showed a significantly higher (1.3-fold) level of these substances in the transgenic cultures overexpressing LEC2.

Taken together, the increased level of indolic compounds found in planta and in vitro in tissue overexpressing LEC2 supports the assumption of the positive impact of LEC2 activity on auxin production. Given that a callus is frequently produced instead of somatic embryos on a standard SE induction medium in response to LEC2 overexpression, we were also interested in determining whether cultures producing a callus differ in auxin content from those developing somatic embryos. Thus, indolic compounds were also compared in Col-0 explants that were efficiently producing somatic embryos and those which failed in SE induction and produced a non-embryogenic callus. The results showed an increased (over 35 %) content of indolic compounds in the callus tissue compared with embryogenic cultures of the same age.

### Expression of auxin biosynthesis-related genes during SE

#### YUCCAs

**Fig. 5:**
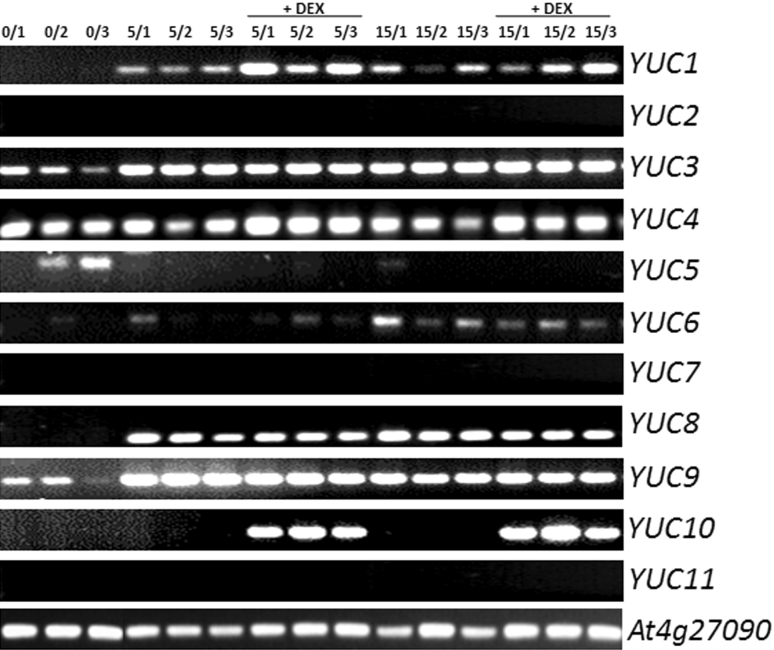
RT-PCR analysis of *YUC* transcripts in embryogenic cultures derived from 35S::LEC2-GR IZE explants on an E5 medium. LEC2 overexpression was induced with DEX (+DEX). RNA samples for the analysis were isolated from 0-, 5-, 15-day-old cultures in three (1–3) independent biological replicates. Culture age/replication number is indicated for each line. *At4g27090* gene encoded 60S ribosomal protein was used as a control for cDNA synthesis

**Fig. 6:**
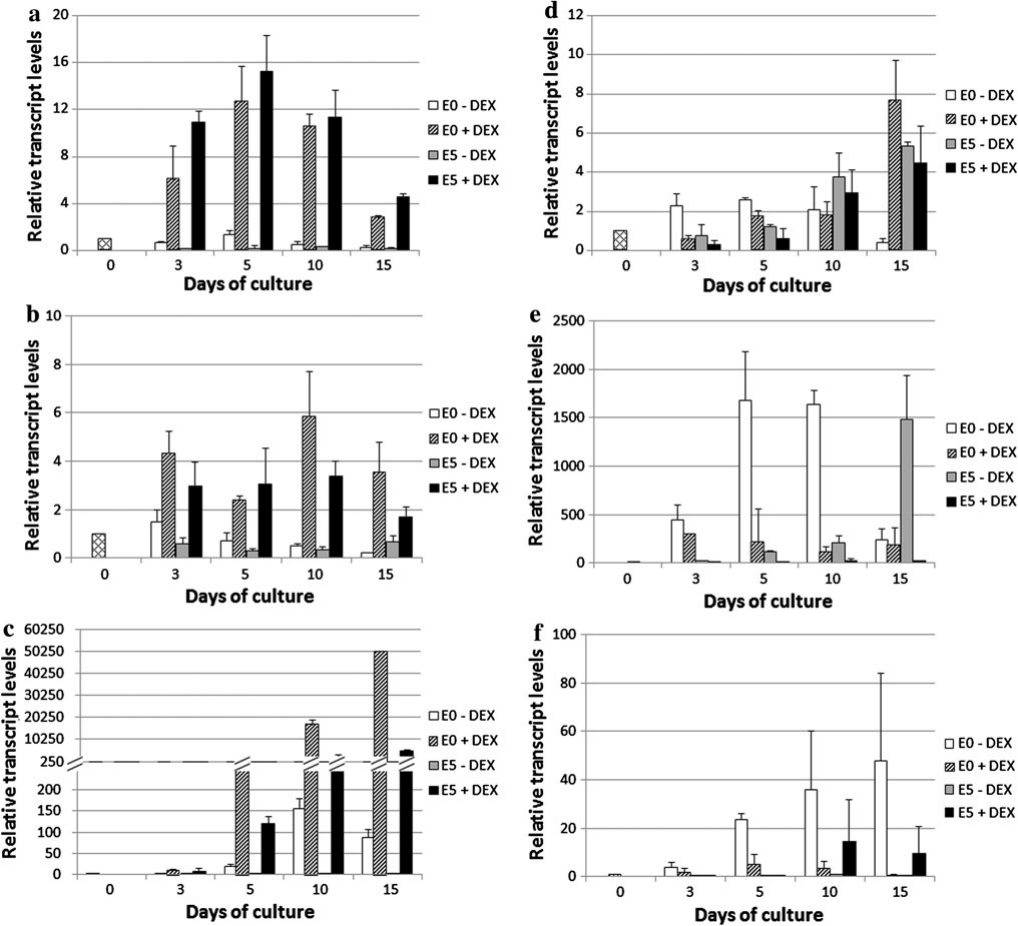
Expression of auxin biosynthesis genes in cultures of 35S::LEC2-GR IZE explants cultured on an auxin-free (E0) and auxin (E5) medium: **a** *YUC1,* **b** *YUC4,* **c** *YUC10,* **d** *TAA1,* **e** *CYP79B2* and **f** *ATR1/MYB34*. *LEC2* overexpression was induced with DEX (+DEX). Bars represent standard deviation (*n* = 3); Relative transcript level was normalised to internal control (*At4g27090*) and calibrated to the 0 day culture

To identify the auxin biosynthesis genes activated in an embryogenic culture in response to LEC2, the expressions patterns of *YUCCA*s, the key components of the main tryptophan-dependent pathway of auxin synthesis in *Arabidopsis*, were analysed in the IZE-derived embryogenic cultures. RT-PCR analysis of all 11 members of the *YUCCA* family in *Arabidopsis* showed transcripts of seven *YUC* genes (*YUC1, 3, 4, 6, 8, 9* and *10*) in the embryogenic culture (Fig. 5). The results suggested that LEC2 stimulated transcription for three of them, *YUC1*, *YUC4* and *YUC10*. To quantify the expression of LEC2-controlled *YUC*s, real-time qRT-PCR analysis was conducted at different stages (0–15 days) of the IZE-derived culture (Fig. 6a–c). This analysis indicated that DEX treatment resulted in the abrupt stimulation of *YUC1* and *YUC4* from an early stage (3 days) of E5-induced cultures, while the expression of *YUC10* increased gradually during SE and the gene was up-regulated over 4,000-fold in a more advanced culture. A significant activation of *YUC1, 4* and *10* in response to LEC2 overexpression was also observed in explants cultured on an auxin-free E0 medium. In addition, a noticeable similarity in the *YUC* expression profiles observed in cultures derived on E5 and E0 media suggests that auxin treatment is rather unlikely to directly affect the transcription of these genes.

In total, the results show that LEC2 seems to stimulate expression of three *YUC* genes (*YUC1, 4* and *10*) in IZE-cultures; however, whether this regulation is direct or not remains unknown. As *LEC2* is highly expressed in a WT embryogenic culture (Ledwoń and Gaj 2009), the involvement of *YUC1, 4* and *10* in the auxin biosynthesis associated with SE induction can therefore be assumed. Because of the notably different expression patterns of *YUC1, 4* and *10* during SE, a different contribution of these genes for SE induction cannot be excluded.

#### TAA1

In addition to the *YUC* genes, the expression pattern of *TRYPTOPHAN AMINOTRANSFERASE OF ARABIDOPSIS*1 (*TAA1*), which is involved in the same tryptophan-dependent pathway of auxin synthesis, was analysed in vitro in relation to LEC2 expression and auxin treatment (Fig. 6d). The results indicate that the high activity of the *TAA1* gene under SE-promoting conditions (DEX-free E5) implies the involvement of this gene in SE induction. In contrast to *YUC* genes, no clear influence of LEC2 activity on *TAA1* expression level was observed in the E5-cultured explants, thus suggesting that the direct control of this gene by LEC2 is rather unlikely.

#### *CYP79B2* and *ATR1/MYB34*

Two other genes, *CYP79B2* and *ATR1/MYB34,* which are involved in the alternative IAOx-mediated tryptophan-dependent pathway of auxin biosynthesis, were analysed in the IZE-derived cultures in relation to the LEC2 transcription level (Fig. 6e, f). In contrast to the *YUC* genes, *CYP79B2* and *ATR1/MYB34* exhibited the highest expression in the DEX- and auxin-free cultures. Moreover, LEC2 overexpression was observed to down-regulate *CYP79B2* transcription regardless of the presence/absence of auxin in a medium. In contrast to the *CYP79B2* and *YUC* genes studied, *ATR1/MYB34* expression in the embryogenic culture of E5-induced explants was found to be extremely low, over 500-fold in comparison to *YUC4,* and no clear influence of LEC2 or auxin on the activity of this gene was observed. Collectively, given that *ATR1/MYB34* is a key activator of IAOx-mediated pathway of auxin biosynthesis, the involvement of this pathway in SE induction seems to be very unlikely.

#### Morphogenic responses of *yucca* mutants

**Fig. 7:**
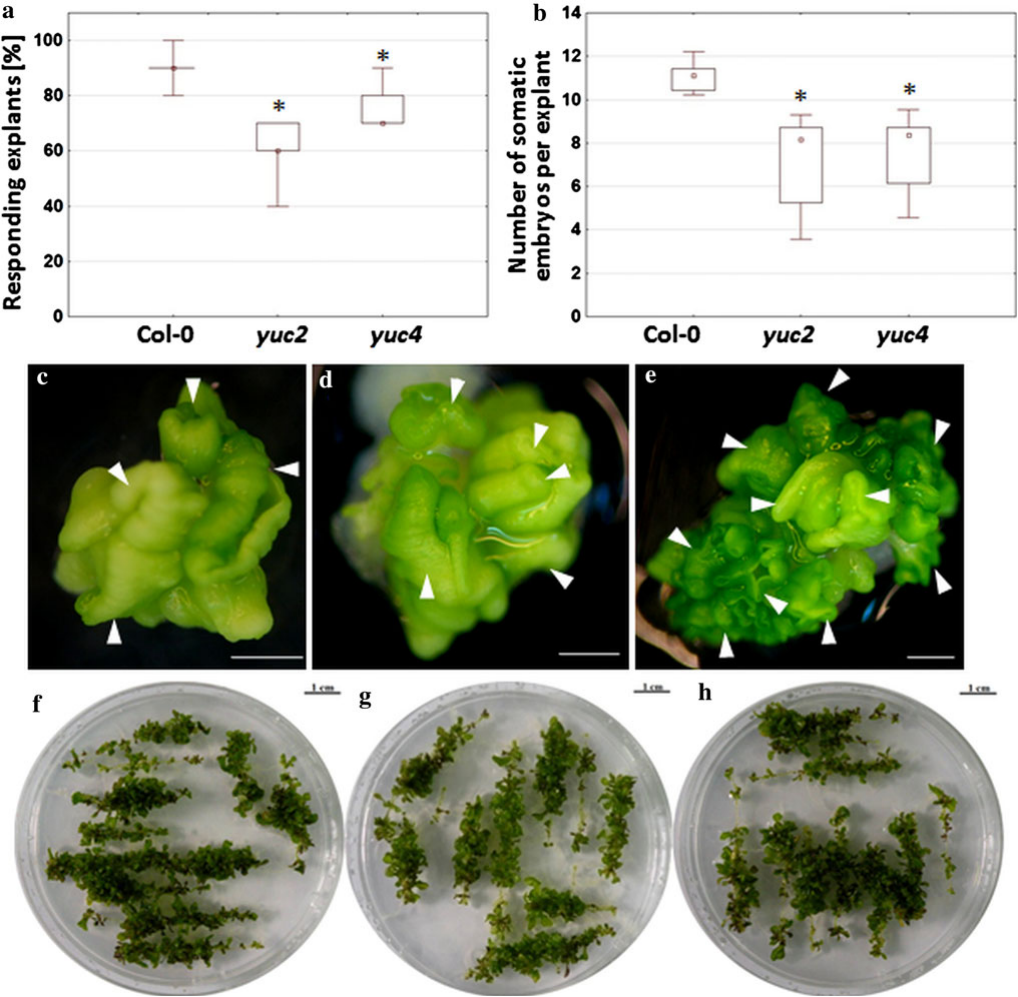
Morphogenic responses of *yuc2* and *yuc4* mutants. Impaired embryogenic capacity of *yuc2* and *yuc4* mutants is manifested by a reduced SE efficiency (**a**) and SE productivity (**b**) of IZE-derived cultures induced on an E5 medium. Reduced number of somatic embryos produced by IZE explant of *yuc2* (**c**) and *yuc4* (**d**) in comparison to Col-0 (**e**). High capacity for shoot organogenesis of root explants of *yuc2* (**f**), *yuc4* (**g**) and Col-0 (**h**) cultured on CIM-SIM-R media. Bars represent standard deviation (*n* = 9). *Scale bar* 1 mm; *Values are significantly different from Col-0 culture

Mutants in two *YUCCA* genes, *yuc2* and *yuc4*, were evaluated in vitro in terms of their capacity for SE and ORG. These knock-out mutations were found to impair the embryogenic response of IZE explants cultured on an E5 auxin medium (Fig. 7a–e). The reduced embryogenic response of the mutants was manifested by a significantly decreased (up to 33 %) frequency of explants producing somatic embryos and more than a 30 % lower number of somatic embryos produced by the mutant explant (Fig. 7a, b). In contrast to SE, an alternative morphogenic pathway, shoot organogenesis, was not affected in the mutant cultures and all of the explants of *yuc2* and *yuc4* mutants developed numerous shoots with productivity comparable to the control (Fig. 7f–h).

## Discussion

The embryogenesis-promoting activity of the LEC2 transcription factor was manifested in planta and in vitro in the vegetative tissue of *Arabidopsis* and somatic embryos were formed on intact LEC2-overexpressing seedlings and IZE explants that were cultured in vitro, respectively (Stone et al. 2001; Ledwoń and Gaj 2009). Therefore, *LEC2* was proposed as providing an embryogenic environment in somatic tissue via an auxin-related mechanism (Stone et al. 2008). In line with this notion, the *YUCCA* genes involved in auxin biosynthesis were reported to be among LEC2 targets (Braybrook et al. 2006; Stone et al. 2008). The SE-promoting effect of LEC2 overexpression was found to be restricted to auxin-free conditions, as exogenously applied auxin was shown to inhibit the embryogenesis-inductive activity of LEC2, possibly through an increasing auxin content or sensitivity (Ledwoń and Gaj 2009).

The present study provides further evidence on the LEC2-mediated positive control of endogenous auxin production during SE induction. The results indicate that LEC2 overexpression reduces the embryogenic potential of explants cultured on auxin media, and regardless of the auxin type, this SE inhibitory effect is related to the auxin concentration in the medium. The negative correlation between the exogenous auxin concentration and SE efficiency observed in 35S::LEC2-GR transgenic culture implied an increase in the auxin content following LEC2 overexpression. A similar conclusion on the positive impact of *LEC2* expression on endogenous auxin was also deduced from the observation that shoot regeneration was replaced by callus formation in the explants overexpressing LEC2 that were cultured under a standard hormone treatment. This callus-promoting response of transgenic explants was found to mimic WT explants induced under high auxin concentrations. The assumption about a positive relation between LEC2 expression and auxin content was further supported, and a significant increase of indolic compounds, including IAA, was indicated in tissue overexpressing LEC2.

For that reason, we aimed to identify the auxin biosynthesis genes that are controlled by LEC2 during SE induction. Three of *YUC* genes (*YUC1, YUC4* and *YUC10)* that are involved in the main tryptophan-dependent auxin biosynthesis pathway were found to be drastically up-regulated by LEC2 in cultured explants. These *YUC* genes are predicted to play essential roles in many developmental processes including ZE (Zhao et al. 2001; Cheng et al. 2007; Zhao 2010). In addition, *YUC4* was expressed in the shoot apex and cotyledon tips of IZE (Cheng et al. 2007), the regions with a higher *LEC2* activity and auxin accumulation, which are involved in the early events that are associated with SE induction (Kurczynska et al. 2007). Thus, it can be speculated that the activity of *YUCs* co-localises with *LEC2* expression and auxin accumulation; however, this remains to be proven. Moreover, further analyses are necessary to reveal whether *YUC* genes are direct transcriptional targets of LEC2 or whether other genetic elements are involved in this interaction.

The activation of several *YUC* genes, including those found to be LEC2-controlled in the present study, was also reported in an embryogenic callus of *Arabidopsis* that was forced to develop somatic embryos via the removal of auxin from a medium (Bai et al. 2012). Collectively, these observations on SE induction in *Arabidopsis* imply that auxin biosynthesis mediated by *YUC* genes is associated with SE induction regardless of the embryogenic system. However, due to the tissue- and developmentally regulated expression of *YUCs* (Cheng et al. 2006), the specific contribution of different *YUC* genes to SE induction in different embryogenic systems remains to be revealed. For example, *YUC2* expression reported in intact *Arabidopsis* seedlings overexpressing LEC2 and forming somatic embryos (Stone et al. 2008) was not found in auxin-induced IZE explants in the present study.

The activity of the *YUCs* in auxin-induced explants cultured in vitro that was observed in the present study parallels the expectation that these genes are regulated by environmental clues, such as hormones and stress factors (Ye et al. 2009). Although specific cis elements related to hormone and stress responses are present in the *YUCs* promoters, the gene regulation mechanisms and the operating transcription factors remain mostly undetermined (Zhao 2010). At present, only *STY/SHI* genes and *LEC2* have been implicated in the regulation of *YUC* expression, and among them, *YUC4* was postulated to be their potential direct target (Sohlberg et al. 2006; Stone et al. 2008). The results of the present study provide further evidence on LEC2-controlled *YUC* activity and indicate that in addition to *YUC4, YUC1* and *YUC10* are also possible targets of this master regulator.

It has become widely accepted that auxin regulates developmental processes via an auxin gradient resulting from a local auxin biosynthesis coupled with polar auxin transport (Kieffer et al. 2010). Auxin polar transport and a local increase in endogenous auxin have been reported in the embryogenic callus cells of *Arabidopsis* that are undergoing embryogenic transition (Su et al. 2009; Bai et al. 2012). The present results support these observations and indicate that LEC2 positively controls the YUC-mediated auxin biosynthesis associated with SE induction.

Despite their tissue- and developmentally specific activity, members of the *YUC* family display overlapping functions (Zhao 2010). Although the inactivation of a single *YUC* gene was reported as not producing any obvious developmental defects in plants (Zhao et al. 2001), we observed a noticeably impaired embryogenic response of individual *yuc2* and *yuc4* mutants. We found that the defective in vitro response of *yucca* mutants was specific for SE, and that the mutated explants retained a high capacity for shoot regeneration. Similarly, *axr4* mutants that were defective in auxin transport (Dharmasiri et al. 2006) were reported to be impaired in their embryogenic response, but not in shoot regeneration via organogenesis (Gaj et al. 2006). Collectively, the SE-specific defects of *yucca* (the present study) and *axr4* (Gaj et al. 2006) mutants confirm that the synthesis and transport of auxin play a key role in SE induction. An appropriate auxin level and gradient seem to be more critical for triggering embryogenic rather than organogenic development. This notion may also explain why a limited spectrum of explants is amenable to somatic embryo induction in contrast to shoot regeneration in most plants (Jiménez 2005). In *Arabidopsis*, the embryogenic capacity of somatic tissue is restricted to one explant type, IZEs, in a strictly defined developmental stage (Gaj 2004), and the increased LEC2 activity in IZE tissue seems to play a central role in SE induction (Ledwoń and Gaj 2009). However, in the present study, we found that LEC2 overexpression is not sufficient for SE induction in explants derived from the post-embryogenic tissue of transgenic seedlings. This observation suggests a highly tissue-specific context of the embryogenesis-promoting activity of LEC2, and that the relevant regulatory mechanism with its genetic components remains to be revealed.

In addition to *YUC* genes, we found *TAA1* to be active during SE induction. *TAA1* acts synergistically with, and upstream to, *YUCs* in the IPA-YUC pathway to enhance IAA biosynthesis in *Arabidopsis* (Mashiguchi et al. 2011; Won et al. 2011). The present analysis showed that the transcription of *TAA1* does not seem to be modulated by LEC2 in an embryogenic culture, but the influence of auxin on gene activity and regulation cannot be excluded.

Our results suggest that in contrast to the IPA-YUC pathway, the alternative indole-3-acetaldoxime (IAOx) pathway of auxin biosynthesis (Mashiguchi et al. 2011) is not involved in SE induction. The analysis of auxin-induced explants indicated an extremely low expression of *ATR1/MYB34*, the key activator in IAOx-mediated auxin synthesis (Celenza et al. 2005). *ATR1/MYB34* and *CYP79B2* of the IPA-YUC pathway have recently been reported to be involved in auxin biosynthesis in the *Arabidopsis* root apex, and interestingly cytokinin was found to be a positive regulator of this pathway (Jones et al. 2010). Thus, it cannot be excluded that the IAOx pathway of auxin synthesis may operate in plants with cytokinin-induced SE systems, although this remains to be explored.

The present study indicates that de novo IAA biosynthesis is associated with SE induction in auxin-treated IZE explants of *Arabidopsis* as was earlier indicated in carrot (Michalczuk et al. 1992), sunflower (Charriere et al. 1999) and alfalfa (Pasternak et al. 2002). Thus, a surge in auxin seems to be a general signal leading to embryogenic transition in the somatic cells of plants. An increase of endogenous auxin in the induction phase of SE was postulated to be triggered by 2,4-D treatment (Fehér et al. 2003), although the exact mechanism remains unknown. Among others, it was proposed that auxin treatment may modulate the endogenous auxin content by affecting the activity of various IAA-related enzymes, such as IAA oxidase and peroxidases (Machakova et al. 2008). Our observations imply that auxin treatment can exert a SE-promoting mechanism via the activation of transcription factors, including LEC2, that control IAA synthesis in cultured tissue. The present work demonstrates the activation of *YUC* genes and an increase of the auxin content in response to an elevated LEC2 activity after auxin treatment. Interestingly, the de novo auxin biosynthesis that is involved in SE induction may also be triggered in response to the removal of auxin from a medium as was recently found in a culture of *Arabidopsis* callus that was forced to develop somatic embryos on an auxin-free medium (Bai et al. 2012). The genetic elements involved in the induction of de novo auxin biosynthesis in different systems of SE induction remain to be uncovered and among them, LEC2 is postulated to play an essential role in directly triggering SE in auxin-treated explant tissue.

**Fig. 8:**
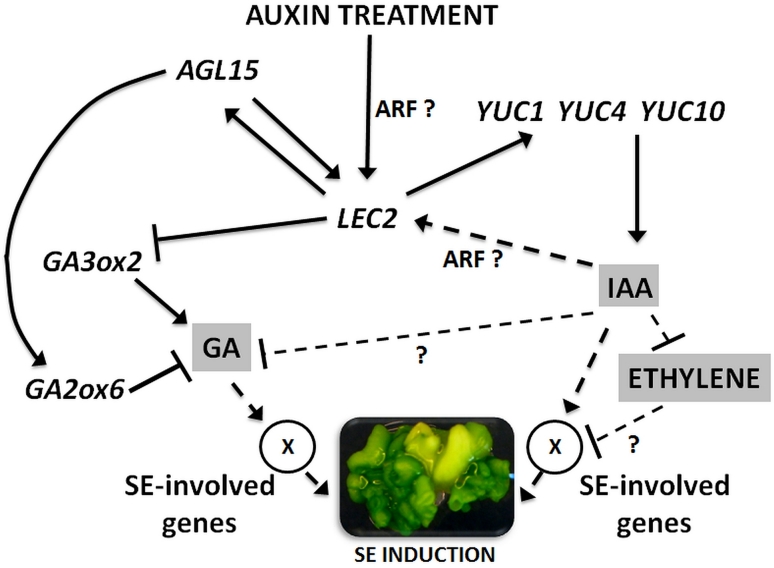
A model for the LEC2 role in SE promotion through gene interactions with auxin, gibberellins (GA) and ethylene. Auxin-stimulated *LEC2* (Ledwoń and Gaj 2009) up-regulates auxin biosynthesis *YUC* genes (the present results). An increase in endogenous IAA activates auxin-responsive SE regulatory genes and results in the inhibition of ethylene biosynthesis and signalling (Bai et al. 2012). LEC2 might also promote SE due to the repression of GA levels via: (1) negative control of *GA3ox2* (Curaba et al. 2004) and (2) positive control of *AGL15* (Braybrook et al. 2006). The interactions that need verification are indicated with *dashed lines*

In conclusion, a possible model of LEC2-induced events associated with embryogenic transition in somatic cells can be proposed in which in addition to auxin other hormones such as gibberellin and ethylene must also be considered (Fig. 8). Further analysis is needed to verify the relations that have been postulated, e.g. between LEC2 and ethylene and to identify other up- and down-stream activated components of the LEC2-controlled pathway that is involved in the induction of embryogenic development in the somatic cells of plants.

## Electronic supplementary material

Below is the link to the electronic supplementary material.

Supplemental **Fig. S1** Characterisation of the *yuc2* and *yuc4* insertional mutants. The insertion in *yuc2* (**a**) and *yuc4* (**b**) mutants results in a knock-out of *YUC2* and *YUC4* genes as indicated in RT-PCR analysis (**c**). *At4g27090* gene encoded 60S ribosomal protein was used as a control for cDNA synthesis (JPG 374 kb)

Supplemental** Fig. S2** Endogenous level of indolic compounds (┬╡g/g of fresh tissue) in different organs of Col-0 (**a**) roots and leaves of seedlings at 10 and 17 DAG, respectively (**b**) old and young leaves of 4-week-old plants. * Values significantly different from DEX-free cultures (JPG 481┬ákb)

## Abbreviations

2,4-D: 2,4-Dichlorophenoxyacetic acid
2iP: 6-(α,α-Dimethylallylamino)-purine
BAP: 6-Benzylaminopurine
CIM: Callus induction medium
DAG: Days after germination
E5: Embryogenesis induction medium with auxin
E0: Auxin-free induction medium
IAA: Indole-3-acetic acid
IZE: Immature zygotic embryo
LEC2: *LEAFY COTYLEDON2*
NAA: 1-Naphthaleneacetic acid
ORG: Shoot organogenesis
SE: Somatic embryogenesis
SIM: Shoot induction medium

## References

[CR1] Angeles-Núnez JG, Tiessen A (2011). Mutation of the transcription factor *LEAFY COTYLEDON2* alters the chemical composition of *Arabidopsis* seeds, decreasing oil and protein content, while maintaining high levels of starch and sucrose in mature seeds. J Plant Physiol.

[CR2] Bai B, Su YH, Yuan J, Zhan XS (2012). Induction of somatic embryos in *Arabidopsis* requires local *YUCCA* expression mediated by the down-regulation of ethylene biosynthesis. Mol Plant.

[CR3] Baud S, Mendoza MS, To A, Harscoet E, Lepiniec L, Dubreucq B (2007). *WRINKLED1* specifies the regulatory action of *LEAFY COTYLEDON2* towards fatty acid metabolism during seed maturation in *Arabidopsis*. Plant J.

[CR4] Boutilier K, Offringa R, Sharma VK, Kieft H, Ouellet T, Zhang L, Hattori J, Liu CM, van Lammeren AAM, Miki BLA, Custers JBM, van Lookeren Compagne MM (2002). Ectopic expression of *BABY BOOM* triggers a conversion from vegetative to embryonic growth. Plant Cell.

[CR5] Bouyer D, Roudier F, Heese M, Andersen ED, Gey D, Nowack MK, Goodrich J, Renou JP, Grini PE, Colot V, Schnittger A (2011). Polycomb repressive complex 2 controls the embryo-to-seedling phase transition. PLoS Genet.

[CR6] Braybrook SA, Harada JJ (2008). *LECs* go crazy in embryo development. Trends Plant Sci.

[CR7] Braybrook SA, Stone SL, Park S, Bui AQ, Le BH, Fischer RL, Goldberg RB, Harada JJ (2006). Genes directly regulated by *LEAFY COTYLEDON2* provide insight into the control of embryo maturation and somatic embryogenesis. Proc Natl Acad Sci USA.

[CR8] Bric JM, Bostock RM, Silverstonet SE (1991). Rapid in situ assay for indoleacetic acid production by bacteria immobilized on a nitrocellulose membrane. Appl Environ Microbiol.

[CR9] Celenza JL, Quiel JA, Smolen GA, Merrikh H, Silvestro AR, Normanly J, Bender J (2005). The *Arabidopsis* ATR1 Myb transcription factor controls indolic glucosinolate homeostasis. Plant Physiol.

[CR10] Charriere F, Sotta B, Miginiac E, Hahne G (1999). Induction of adventitious shoots or somatic embryos on in vitro cultured zygotic embryos of *Helianthus annuus*: variation of endogenous hormone levels. Plant Physiol Biochem.

[CR11] Cheng Y, Dai X, Zhao Y (2006). Auxin biosynthesis by the YUCCA flavin monooxygenases controls the formation of floral organs and vascular tissues in *Arabidopsis*. Genes Dev.

[CR12] Cheng Y, Dai X, Zhao Y (2007). Auxin synthesized by the YUCCA flavin monooxygenases is essential for embryogenesis and leaf formation in *Arabidopsis*. Plant Cell.

[CR13] Curaba J, Moritz T, Blervaque R, Parcy F, Raz V, Herzog M, Vachon G (2004). *AtGA3ox2*, a key gene responsible for bioactive gibberellin biosynthesis, is regulated during embryogenesis by *LEAFY COTYLEDON2* and *FUSCA3* in *Arabidopsis*. Plant Physiol.

[CR14] Dharmasiri S, Swarup R, Mockaitis K, Dharmasiri N, Singh SK, Kowalchyk M, Marchant A, Mills S, Sandberg G, Bennett MJ, Estelle M (2006). AXR4 is required for localization of the auxin influx facilitator AUX1. Science.

[CR15] Fehér A, Pasternak TP, Dudits D (2003). Transient of somatic plant cells to an embryogenic state. Plant Cell Tiss Org Cult.

[CR16] Feldmann KA, Marks MD (1986). Rapid and efficient regeneration of plants from explants of *Arabidopsis thaliana*. Plant Sci.

[CR17] Gaj MD (2001). Direct somatic embryogenesis as a rapid and efficient system for in vitro regeneration of *Arabidopsis thaliana*. Plant Cell Tiss Org Cult.

[CR18] Gaj MD (2004). Factors influencing somatic embryogenesis induction and plant regeneration with particular reference to *Arabidopsis thaliana* (L.) Heynh. Plant Growth Regul.

[CR19] Gaj MD, Zhang S, Harada JJ, Lemaux PG (2005). *LEAFY COTYLEDON* genes are essential for induction of somatic embryogenesis of *Arabidopsis*. Planta.

[CR20] Gaj MD, Trojanowska A, Ujczak A, Mędrek M, Kozioł A, Garbaciak B (2006). Hormone-response mutants of *Arabidopsis thaliana* (L.) Heynh. impaired in somatic embryogenesis. Plant Growth Regul.

[CR21] Gamborg OL, Miller RA, Ojima K (1968). Nutrient requirement of suspension culture of soybean root cells. Exp Cell Res.

[CR22] Graaff E, Boot K, Granbom R, Sandberg G, Hooykaas PJJ (2003). Increased endogenous auxin production in *Arabidopsis thaliana* causes both earlier described and novel auxin-related phenotypes. J Plant Growth Regul.

[CR23] Harding EW, Tang W, Nichols KW, Fernandez DE, Perry SE (2003). Expression and maintenance of embryogenic potential is enhanced through constitutive expression of *AGAMOUS*-*LIKE15*. Plant Physiol.

[CR24] Imin N, Goffard N, Nizamidin M, Rolfe BG (2008). Genome-wide transcriptional analysis of super-embryogenic *Medicago truncatula* explant cultures. BMC Plant Biol.

[CR25] Jiménez VM (2005). Involvement of plant hormones and plant growth regulators on in vitro somatic embryogenesis. Plant Growth Regul.

[CR26] Jones B, Gunneras SA, Petersson SV, Tarkowski P, Graham N, May S, Dolezal K, Sandberg G, Ljung K (2010). Cytokinin regulation of auxin synthesis in *Arabidopsis* involves a homeostatic feedback loop regulated via auxin and cytokinin signal transduction. Plant Cell.

[CR27] Kieffer M, Neve J, Kepinski S (2010). Defining auxin response contexts in plant development. Curr Opin Plant Biol.

[CR28] Kowalczyk M, Sandberg G (2001). Quantitative analysis of indole-3-acetic acid metabolites in *Arabidopsis*. Plant Physiol.

[CR29] Kraut M, Wójcikowska B, Ledwoń A, Gaj MD (2011). Immature zygotic embryo cultures of *Arabidopsis*—a model system for molecular studies on morphogenic pathways induced in vitro. Acta Biol Cracov Bot.

[CR30] Kroj T, Savino G, Valon C, Giraudat J, Parcy F (2003). Regulation of storage protein gene expression in *Arabidopsis*. Development.

[CR31] Kurczynska EU, Gaj MD, Ujczak A, Mazur E (2007). Histological analysis of direct somatic embryogenesis in *Arabidopsis thaliana* (L.) Heynh.. Planta.

[CR32] Ledwoń A, Gaj MD (2009). *LEAFY COTYLEDON2* gene expression and auxin treatment in relation to embryogenic capacity of *Arabidopsis* somatic cells. Plant Cell Rep.

[CR33] Lucau-Danila A, Laborde L, Legrand S, Huot L, Hot D, Lemoine Y, Hilbert JL, Hawkins S, Quillet MC, Hendriks T, Blervacq AS (2010). Identification of novel genes potentially involved in somatic embryogenesis in chicory (*Cichorium intybus* L.). BMC Plant Biol.

[CR34] Luo S, Wan Y, Xiao X, Guo H, Chen L, Xi Q, Zeng G, Liu C, Chen J (2011). Isolation and characterization of endophytic bacterium LRE07 from cadmium hyperaccumulator *Solanum nigrum* L. and its potential for remediation. Appl Microbiol Biotechnol.

[CR35] Ma Y, Rajkumar M, Freitas H (2009). Isolation and characterization of Ni mobilizing PGPB from serpentine soils and their potential in promoting plant growth and Ni accumulation by *Brassica* spp.. Chemosphere.

[CR36] Machakova I, Zazimalova E, George EF, George EF, Hall MA, De Klerk GJ (2008). Plant growth regulators I: introductions, auxins, their analogous and inhibitors. Plant propagation by tissue culture.

[CR37] Mashiguchi K, Tanaka K, Sakai T, Sugawara S, Kawaide H, Natsume M, Hanada A, Yaeno T, Shirasu K, Yao H, McSteen P, Zhao Y, Hayashi KI, Kamiya Y, Kasahara H (2011). The main auxin biosynthesis pathway in *Arabidopsis*. Proc Natl Acad Sci USA.

[CR38] Meinke DW, Franzmann LH, Nickle TC, Yeung EC (1994). *Leafy cotyledon* mutants of *Arabidopsis*. Plant Cell.

[CR39] Michalczuk L, Cooke TJ, Cohen JD (1992). Auxin levels at different stages of carrot somatic embryogenesis. Phytochemistry.

[CR40] Murashige T, Skoog FA (1962). A revised medium for rapid growth and bioassays with tobacco tissue cultures. Physiol Plant.

[CR41] Ogas J, Kaufmann S, Henderson J, Somerville C (1999). PICKLE is a CHD3 chromatin-remodeling factor that regulates the transition from embryonic to vegetative development in *Arabidopsis*. Proc Natl Acad Sci USA.

[CR42] Pasternak TP, Prinsen E, Ayaydin F, Miskolczi P, Potters G, Asard H, Van Onckelen HA, Dudits D, Fehér A (2002). The role of auxin, pH, and stress in the activation of embryogenic cell division in leaf protoplast-derived cells of alfalfa. Plant Physiol.

[CR43] Sablowski RWM, Meyerowitz EM (1998). A homolog of *NO APICAL MERISTEM* is an immediate target of the floral homeotic genes *APETALA3/PISTILLATA*. Cell.

[CR44] Santos-Mendoza M, Dubreucq B, Miquel M, Caboche M, Lepiniec L (2005). *LEAFY COTYLEDON2* activation is sufficient to trigger the accumulation of oil and seed specific mRNAs in *Arabidopsis* leaves. FEBS Lett.

[CR45] Sharma SK, Millam S, Hedley PE, McNicol J, Bryan GJ (2008). Molecular regulation of somatic embryogenesis in potato: an auxin led perspective. Plant Mol Biol.

[CR46] Sohlberg JJ, Myrenas M, Kuusk S, Lagercrantz U, Kowalczyk M, Sandberg G, Sundberg E (2006). *STY1* regulates auxin homeostasis and affects apical–basal patterning of the *Arabidopsis* gynoecium. Plant J.

[CR47] Stone SL, Kwong LW, Yee KM, Pelletier J, Lepiniec L, Fischer RL, Goldberg RB, Harada JJ (2001). *LEAFY COTYLEDON2* encodes B3 domain transcription factor that induces embryo development. Proc Natl Acad Sci USA.

[CR48] Stone SL, Braybrook SA, Paula SL, Kwong LW, Meuser J, Pelletier J, Hsieh TF, Fischer RL, Goldberg RB, Harada JJ (2008). *Arabidopsis LEAFY COTYLEDON2* induces maturation traits and auxin activity: implications for somatic embryogenesis. Proc Natl Acad Sci USA.

[CR49] Su YH, Zhao XY, Liu YB, Zhang CL, O’Neill SD, Zhang XS (2009). Auxin-induced *WUS* expression is essential for embryonic stem cell renewal during somatic embryogenesis in *Arabidopsis*. Plant J.

[CR50] Thellin O, Zorzi W, Lakaye B, De Borman B, Coumans B, Hennen G, Grisar T, Igout A, Heinen E (1999). Housekeeping genes as internal standards: use and limits. J Biotechnol.

[CR51] Thibaud-Nissen F, Shealy RT, Khanna A, Vodkin LO (2003). Clustering of microarray data reveals transcript patterns associated with somatic embryogenesis in soybean. Plant Physiol.

[CR52] Tsuwamoto R, Yokoi S, Takahata Y (2010). *Arabidopsis**EMBRYOMAKER* encoding an AP2 domain transcription factor plays a key role in developmental change from vegetative to embryonic phase. Plant Mol Biol.

[CR53] Wang X, Niu QW, Teng C, Li C, Mu J, Chua NH, Zuo J (2009). Overexpression of *PGA37/MYB118* and *MYB115* promotes vegetative-to-embryonic transition in *Arabidopsis*. Cell Res.

[CR54] Wang W, Yang J, Liu H, Lu D, Chen X, Zenonos Z, Campos LS, Rad R, Guo G, Zhang S, Bradley A, Liu P (2011). Rapid and efficient reprogramming of somatic cells to induced pluripotent stem cells by retinoic acid receptor gamma and liver receptor homolog 1. Proc Natl Acad Sci USA.

[CR55] Won C, Shen X, Mashiguchi K, Zheng Z, Dai X, Cheng Y, Kasahara H, Kamiya Y, Chory J, Zhao Y (2011). Conversion of tryptophan to indole-3-acetic acid by *TRYPTOPHAN AMINOTRANSFERASES OF ARABIDOPSIS* and YUCCAs in *Arabidopsis*. Proc Natl Acad Sci USA.

[CR56] Ye X, Kang BG, Osburn LD, Li Y, Zong-Ming C (2009). Identification of the flavin-dependent monooxygenase-encoding *YUCCA* gene family in *Populus trichocarpa* and their expression in vegetative tissues and in response to hormone and environmental stresses. Plant Cell Tiss Org Cult.

[CR57] Zhao Y (2010). Auxin biosynthesis and its role in plant development. Annu Rev Plant Biol.

[CR58] Zhao Y, Christensen SK, Fankhauser C, Cashman JR, Cohen JD, Weigel D, Chory J (2001). A role for flavin monooxygenase-like enzymes in auxin biosynthesis. Science.

[CR59] Zuo J, Niu QW, Frugis G, Chua NH (2002). The *WUSCHEL* gene promotes vegetative-to-embryonic transition in *Arabidopsis*. Plant J.

