# Supplementary figures and images for: LEAFY COTYLEDON2 (LEC2) promotes embryogenic induction in somatic tissues of Arabidopsis, via YUCCA-mediated auxin biosynthesis
Source: Planta. 2013 May 31;238(3):425–40. doi: 10.1007/s00425-013-1892-2 (PMC3751287; doi:10.1007/s00425-013-1892-2)

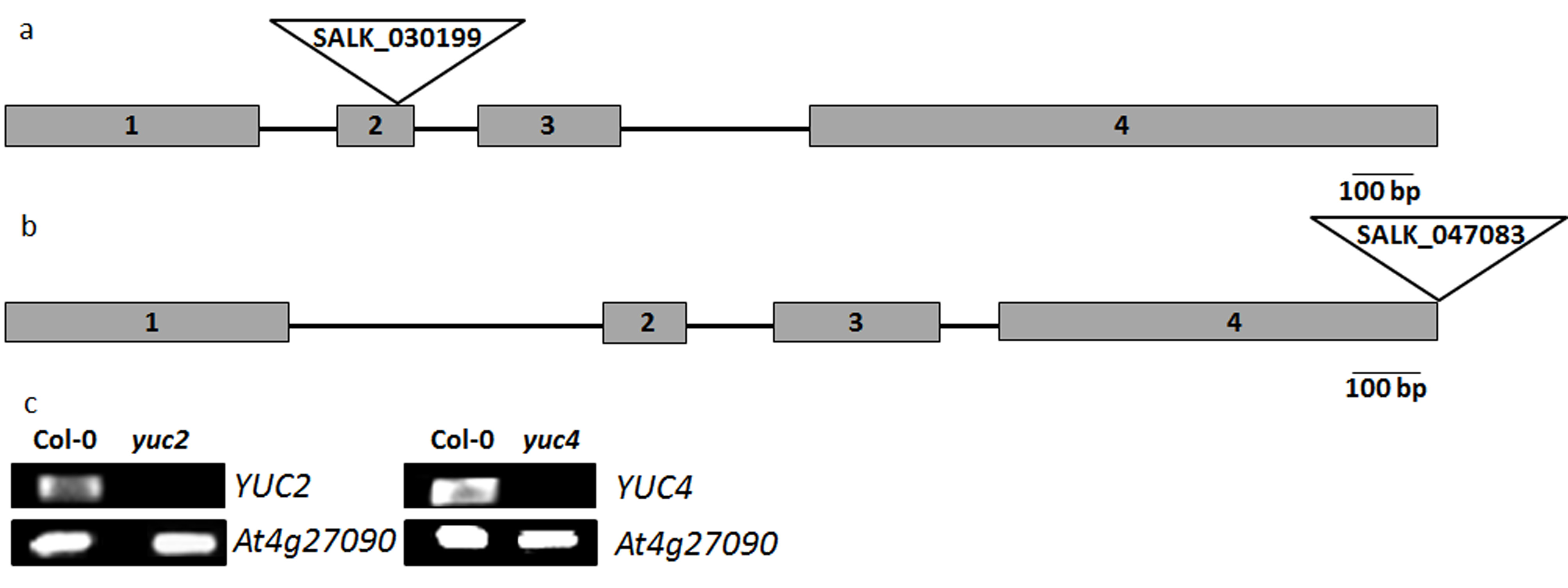

Supplement: Supplementary file 1 — Supplemental Fig. S1 Characterisation of the yuc2 and yuc4 insertional mutants. The insertion in yuc2 (a) and yuc4 (b) mutants results in a knock-out of YUC2 and YUC4 genes as indicated in RT-PCR analysis (c). At4g27090 gene encoded 60S ribosomal protein was used as a control for cDNA synthesis (JPG 374 kb) [file 425_2013_1892_MOESM1_ESM.jpg]

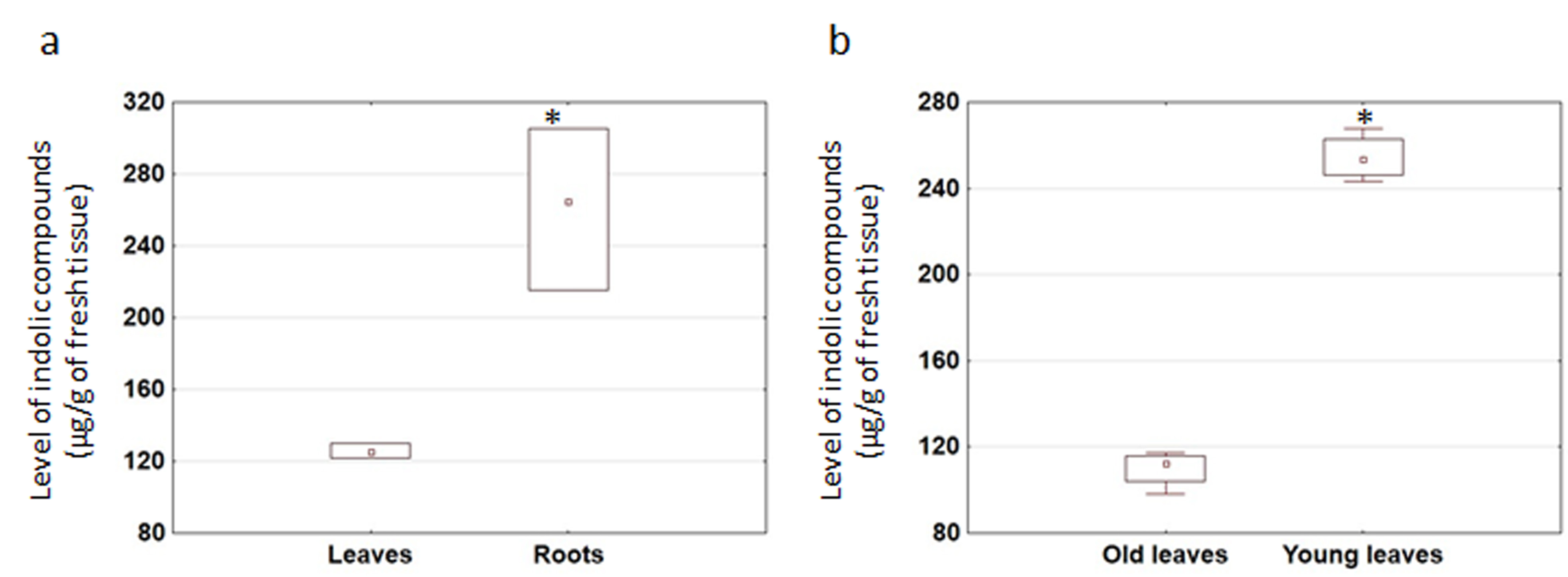

Supplement: Supplementary file 2 — Supplemental Fig. S2 Endogenous level of indolic compounds (┬╡g/g of fresh tissue) in different organs of Col-0 (a) roots and leaves of seedlings at 10 and 17 DAG, respectively (b) old and young leaves of 4-week-old plants. * Values significantly different from DEX-free cultures (JPG 481┬ákb) [file 425_2013_1892_MOESM2_ESM.jpg]
